# Prevalence and associated factors of schistosomiasis among children and adolescents visiting Chitokoloki Mission Hospital of Zambezi District

**DOI:** 10.1186/s12879-025-12267-6

**Published:** 2025-12-01

**Authors:** Martin Chakulya, David Chisompola, Nyondo Chawela, Hanzooma Hatwiko, Matenge Mutalange, Memory Ngosa, Geofrey Mupeta, Marshall C. Mubanga, Lukundo Siame, Chipego Hajamba, Ebenezer Banda, Joreen P. Povia, Nzooma M. Shimaponda-Mataa, Sepiso K. Masenga

**Affiliations:** 1 Department of Pathology and Microbiology, School of Medicine and Health Sciences, Mulungushi University, Livingstone, Zambia; 2 Department of Pathology, Arthur Division Children’s Hospital, Ndola, Zambia; 3 Department of Biomedical Sciences, University of Zambia, Lusaka, Zambia; 4 Department of Cardiovascular Science and Metabolic Diseases, Livingstone Center for Prevention and Translational Science, Livingstone, Zambia

**Keywords:** Children, Prevalence, Schistosomiasis, Urogenital infection, Zambia

## Abstract

**Background:**

Urogenital schistosomiasis remains a major public health challenge among children and adolescents in sub-Saharan Africa. Data on prevalence and associated factors in Zambia are scarce. This study assessed the burden of *Schistosoma haematobium* infection and its correlates among 271 children and adolescents attending the outpatient department of Chitokoloki Mission Hospital, Northwestern Province.

**Methods:**

We conducted a retrospective cross-sectional study of clinical records from January to March 2025. Systematic random sampling of files for participants aged 5–18 years captured sociodemographic data, water-contact behaviours, haematuria, haematological indices (haemoglobin, MCV, MCHC), deworming history, and prior schistosomiasis. Urine microscopy for *S. haematobium* eggs defined infection status. Categorical variables were compared by chi-squared test and medians by Wilcoxon rank-sum. Multivariable logistic regression identified independent predictors of infection (*p* < 0.05).

**Results:**

The median age was 13 years (IQR: 12–15); 58.3% were male. Overall prevalence of schistosomiasis was 25.8% (*n* = 70). Haematuria was present in 80.2% of infected versus 0.5% of uninfected participants (*p* < 0.0001). Infected children had lower median haemoglobin (11.3 g/dL vs. 11.9 g/dL; *p* = 0.0067) and MCHC (30.9 g/dL vs. 32.1 g/dL; *p* = 0.0022). Only 3.6% of previously dewormed children were infected compared with 49.2% of non-dewormed peers (*p* < 0.0001). In adjusted odds ratio (aOR) analyses, absence of deworming (aOR 37.8; 95% CI 5.78–247.4), absence of haematuria (aOR 0.0014; 95% CI 0.0001–0.011), and lower haemoglobin (aOR 0.69 per g/dL; 95% CI 0.48–0.99) remained independently associated with infection.

**Conclusions:**

There is a significant burden of urogenital schistosomiasis among school-aged children and adolescents attending Chitokoloki Mission Hospital in Zambezi District, Zambia. Targeted praziquantel distribution, cost-effective school-based urine screening in high endemic areas, awareness campaigns to sensitize the community on transmission and reinfections and the integration of nutritional and anaemia management strategies are critical components for effective disease control. Strengthening these interventions is essential to advance progress toward achieving the World Health Organization’s 2030 schistosomiasis elimination targets in Zambia.

**Clinical trial number:**

Not applicable (N/A).

## Introduction

Schistosomiasis, a debilitating parasitic disease caused by blood flukes of the genus Schistosoma, remains a critical public health challenge in tropical and subtropical regions worldwide [1]. Transmission occurs primarily through skin contact with freshwater contaminated by cercariae released from infected intermediate snail hosts, such as Bulinus truncatus for *Schistosoma haematobium* [2, 3]. Globally, over 240 million people are infected annually, resulting in approximately 280,000 deaths and accounting for more than 3.3 million disability-adjusted life years, ranking it among the most devastating neglected tropical diseases [4, 5]. The burden of *Schistosoma haematobium* is disproportionately concentrated in sub-Saharan Africa (SSA), where impoverished communities face cyclical reinfection due to inadequate water, sanitation, and hygiene (WASH) infrastructure [6–8].

Children and adolescents bear the highest burden of schistosomiasis morbidity. Their frequent exposure to contaminated water during daily activities like swimming, bathing, fetching water, and fishing significantly increases infection risk [7, 9, 10]. The prevalence of schistosomiasis in this demographic can exceed 75% in high-transmission foci [11, 12]. Chronic infection during critical developmental stages leads to severe health complications, including hepatic fibrosis, portal hypertension, kidney failure, bladder cancer, and urogenital damage [13, 14]. Beyond physical pathology, schistosomiasis causes anaemia, malnutrition, stunted growth, and impaired cognitive development, profoundly impacting educational attainment and long-term quality of life [15, 16]. Despite global control efforts, including preventative chemotherapy targeting millions, prevalence remains persistently high, with an estimated 251.4 million people requiring treatment in 2022. Current strategies often fail to adequately address the specific vulnerabilities and transmission dynamics affecting youth [17, 18].

In Zambia, schistosomiasis is endemic yet critically understudied, particularly among children and adolescents. Reported prevalence of approximately 9.7%-35.5% indicates substantial transmission [19, 20]. The surge in praziquantel uptake at facilities like Chitokoloki Mission Hospital underscores a significant local burden. However, recent and comprehensive data on the prevalence, severity, geographic distribution, and key risk factors of schistosomiasis among children and adolescents in Zambia remain limited. Understanding these correlates including environmental, socioeconomic, behavioral, and WASH-related factors is essential for designing effective, targeted interventions.

Mass drug administration (MDA) with praziquantel among children has been implemented in Zambia like in many other SAA as a key control strategy in highly endemic areas for schistosomiasis. Despite these efforts, the country has not yet achieved the WHO target of attaining at least 75% treatment coverage among at-risk school-aged children [20]. The effectiveness of the MDA program is further undermined by frequent reinfections resulting from continued exposure to infested water sources, such as rivers, dams, and ponds, which serve as the main habitats for intermediate snail hosts [21]. Moreover, challenges such as inadequate community sensitization, irregular drug supply, and limited integration of WASH interventions have contributed to the persistence of transmission and hindered the long-term impact of praziquantel-based control programs [22]. Therefore, it remains unclear whether the current control efforts are sufficiently effective, highlighting the need to re-evaluate and strengthen existing schistosomiasis control strategies in Zambia.

This study therefore aims to assess the burden and identify the correlates of schistosomiasis among children and adolescents in Zambia. By quantifying prevalence and analyzing determinants of infection, this research will generate critical evidence to inform local and national public health strategies.

## Methodology

### Study design and study setting

This was a retrospective cross-sectional study conducted at Chitokoloki Mission Hospital in North-western Province, Zambia, among children and adolescents attending the outpatient department between 1 January 2025 and 31 March 2025. Chitokoloki Mission Hospital is a 180–200 bed capacity healthcare facility located in Zambezi District, Zambia. It serves a catchment population of approximately 150,000 people. In addition to providing inpatient and outpatient services, the hospital supports three satellite clinics within a 40 km radius, two of which are situated on the west bank of the Zambezi River, ensuring access to healthcare for remote and underserved communities. The outpatient department serves as the primary referral point for surrounding health centres in the district.

### Eligibility and recruitment criteria

**Fig. 1:**
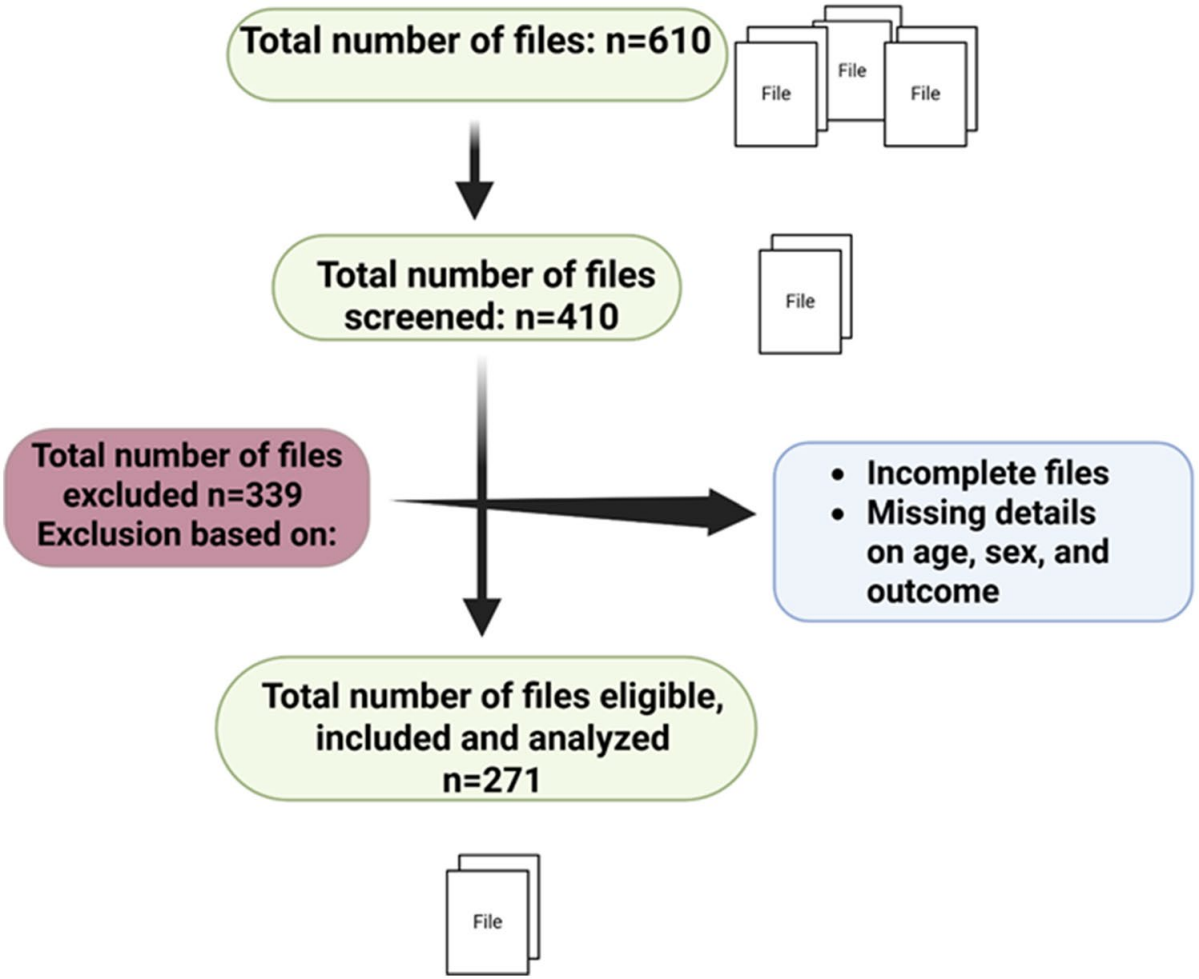
Participant selection flow chart

We abstracted hospital records from participants aged 5 to 18 years who visited the outpatient department. Records missing clinical or laboratory data were excluded. Using a systematic random sampling approach, every third file from the eligible patient records was selected for screening. From 610 files available for abstraction, 410 medical records were reviewed, 271 records met the inclusion criteria and were included in the analysis. To ensure data completeness, each record underwent a structured verification checklist confirming availability of demographic, clinical, and laboratory variables before inclusion. A total of 339 records were excluded due to incomplete information, such as missing details on age, sex, and missing outcome, diagnosis or the outcome, Fig. 1. Quality control checks were conducted by a second reviewer who independently verified the included records to ensure accuracy and consistency of abstraction.

### Sample size calculations

The sample size for this study was determined using the single population proportion formula, based on an estimated prevalence of 20.3% [23], a 95% confidence level, and a margin of error of 5%. The formula applied was:

$$\:n=\frac{{Z}^{2}\times\:p\times\:(1-p)}{{d}^{2}}$$

where n is the required sample size, Z is the Z-score corresponding to a 95% confidence level (1.96), p is the estimated prevalence (0.203), and d is the desired margin of error (0.05). Substituting these values into the formula yielded a minimum sample size of approximately 248. To account for potential non-response or incomplete data, a 10% adjustment was made, resulting in a final sample size of 271 participants included in the study.

### Specimen collection and processing 

As this was a retrospective study, all biological specimens had been collected and processed as part of routine clinical procedures at Chitokoloki Mission Hospital. According to standard hospital protocol, patients were provided with sterile urine containers and instructed to collect midstream urine samples to minimize contamination. The initial urine stream was discarded, and only the midstream portion was retained for analysis. At the same clinical visit, approximately 4 mL of venous blood was aseptically drawn from the antecubital fossa using EDTA vacutainer tubes. All samples were labeled with unique patient identifiers at the point of collection and transported to the laboratory in cooler boxes for testing maintained at recommended temperatures. Haematological analysis was conducted using a Horiba Micros ES60 haematology analyzer (Horiba ABX, Montpellier, France) to assess complete blood count (CBC) parameters, including haemoglobin concentration, mean corpuscular volume (MCV), mean corpuscular haemoglobin (MCH), and mean corpuscular haemoglobin concentration (MCHC). Urine samples were initially inspected for visible blood (macrohaematuria), followed by dipstick testing using Hemastix reagent strips (Siemens Healthcare Diagnostics) to detect microhaematuria. Microscopic examination for *Schistosoma haematobium* eggs was performed on centrifuged urine sediments using standard light microscopy techniques [24]. Urine microscopy for *Schistosoma haematobium* eggs was performed exclusively by two trained, licensed and experienced laboratory technologists following the standard operating procedures. Inter-observer reliability was adhered to and any discrepancies between the two investigators were resolved by consensus.

### Data collection

Clinical and demographic data for 271 children and adolescents (aged 5–18 years) attending the outpatient department at Chitokoloki Mission Hospital in North-western Province, Zambia, were abstracted from both paper and electronic records. Key variables including age, sex, access to clean water, primary source of drinking water, water‑contact activities (swimming, washing clothes, drawing water), haematuria, complete blood count indices including haemoglobin, MCV, MCH, MCHC, differential leukocyte counts, platelets and deworming history were captured into REDCap. Records lacking essential clinical or laboratory data were excluded. Each file was screened and selected via systematic random sampling, with every third eligible record abstracted for analysis. Data completeness was validated through double-entry checks, and inconsistencies were resolved by referring back to the original source documents.

### Study variables

The primary outcome was schistosomiasis infection, defined as the presence of Schistosoma *haematobium* eggs on urine microscopy. Independent variables included demographic factors (age, sex), water‑related exposures (access to clean water, primary drinking‑water source, proximity to and activities in streams), clinical markers (haematuria, haemoglobin concentration, MCV, MCH, MCHC), and prevention history (praziquantel deworming status, self‑reported prior schistosomiasis).

### Data analysis

Following data abstraction into REDCap, the data were exported to Microsoft Excel for cleaning and subsequently analyzed in StatCrunch. Categorical variables were summarized using frequencies and percentages, while continuous variables were described as medians with interquartile ranges (IQRs). The Shapiro–Wilk test assessed normality, and the Wilcoxon rank-sum test compared medians between infected and uninfected groups. Associations between categorical variables and schistosomiasis status were evaluated using chi‑squared tests. Factors associated with schistosomiasis were then examined via multivariable logistic regression. Candidate predictors including age, sex, access to clean water, primary drinking‑water source, water‑contact activities such as swimming, drawing water, haematuria, haemoglobin concentration, and deworming history were chosen based on prior literature and field expert input. Variables with a univariate *p* < 0.05 or established biological relevance were retained in the adjusted model. Statistical significance was defined as *p* < 0.05.

### Ethical considerations

All extracted data were anonymized prior to analysis by removing personal identifiers and assigning unique study codes. Data were stored in password-protected files accessible only to the study team. Ethical approval and data-protection procedures were aligned with the declarations described in the ethics section.

## Results

**Fig. 2:**
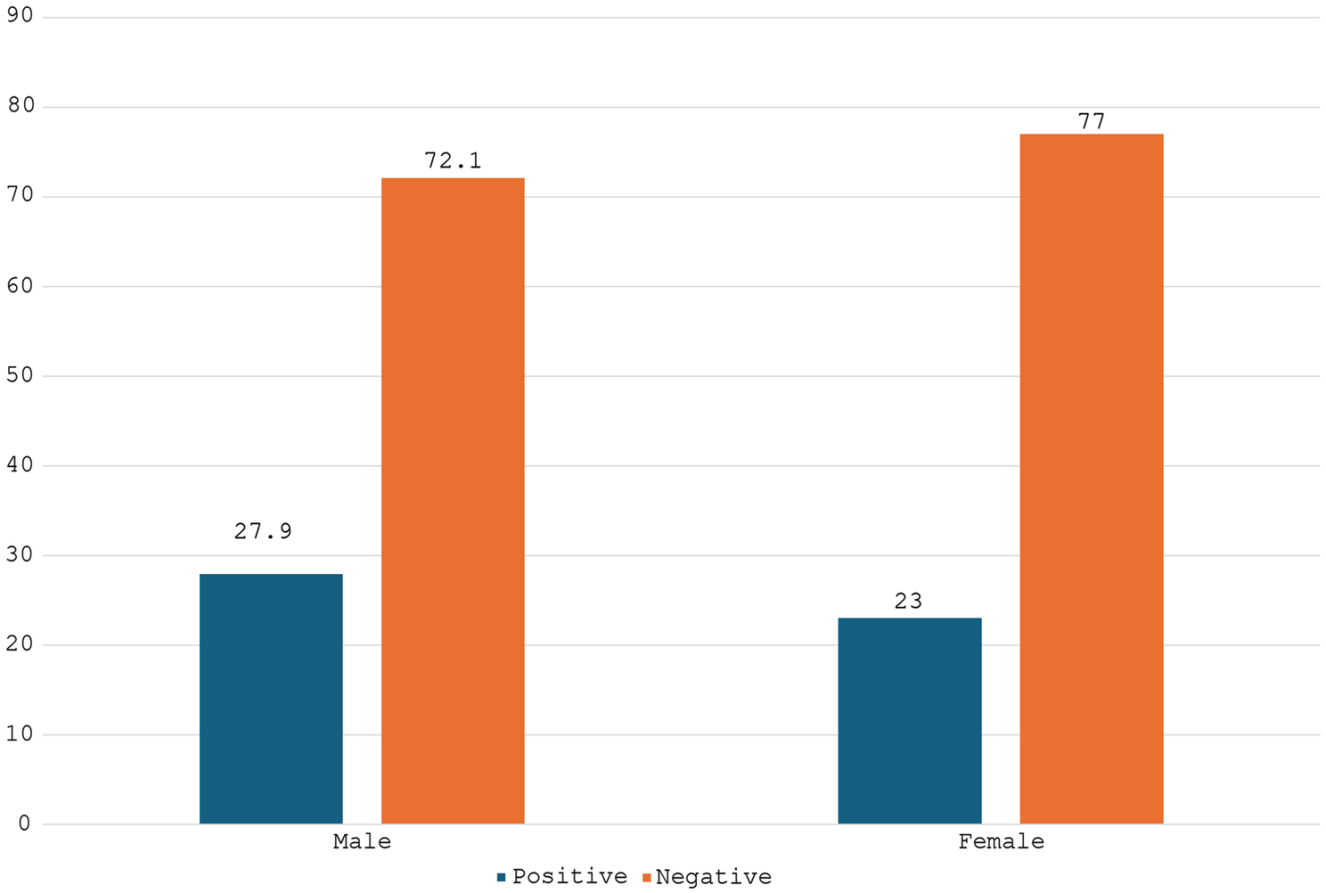
Prevalence of schistosomiasis by sex

The study included 271 participants with a median age of 13 years (interquartile range [IQR]: 12–15), of whom 25.8% (95% CI: 20.8%–31.5%; *n* = 70) had schistosomiasis (Table 1). Most participants were male (58.3%, *n* = 158), used river water as their primary drinking source (60.5%, *n* = 164), lived near streams (85.6%, *n* = 232), and swam in streams (96.3%, *n* = 261). Prevalence estimates by sex were 27.9% (95% CI: 21.4%–35.6%) among males and 23.0% (95% CI: 16.0%–31.6%) among females (Fig. 2). Schistosomiasis prevalence showed no significant associations with sex (*p* = 0.370), access to clean water (*p* = 0.645), water source (*p* = 0.618), symptoms (*p* = 0.902), proximity to streams (*p* = 0.412), or water-contact activities (e.g., swimming: *p* = 0.722) (Table 1). Significant findings included: a strong association between haematuria and infection (80.2% [69/86] vs. 0.5% [1/185]; *p* < 0.0001), lower haemoglobin levels in infected participants (median: 11.3 g/dL, IQR: 8.3–12.3 vs. 11.9 g/dL, IQR: 10.3–13.5; *p* = 0.0067), and reduced mean corpuscular haemoglobin concentration (MCHC) (30.9 g/dL vs. 32.1 g/dL; *p* = 0.0022). Deworming (3.6% [5/139] infected vs. 49.2% [65/132]; *p* < 0.0001) and prior schistosomiasis history (9.9% [7/71] vs. 31.5% [63/200]; *p* < 0.0001) were strongly protective (Table 1).

Missing data were handled using listwise deletion, whereby participants with incomplete data for a given analysis were excluded from that specific statistical test. The overall proportion of missing data was minimal and did not affect the robustness of the results.

**Table 1:** General characteristics of study participants

Variable	Median (IQR) /frequency (%)	Schistosomiasis		*P* value
		Pos (+) = 70 (25.8)	Neg (-) = 201 (74.2)	
**Age years**	13 (12, 15)	13 (12,15)	13 (12,15)	0.4272
**Sex**				
* Male*	158 (58.3)	44 (27.9)	114 (72.2)	0.370
* Female*	113 (41.7)	26 (23.0)	87 (77.0)	
**Access to clean water**				
* Yes*	106 (39.1)	29 (27.4)	77 (72.6)	0.645
* No*	165 (60.9)	41 (24.9)	124 (75.1)	
**Source of drinking water**				
* Tap*	105 (38.8)	29 (27.6)	76 (72.4)	0.618
* River water*	164 (60.5)	40 (41.8)	124 (75.6)	
**Any signs and symptoms**				
* Yes*	99 (36.5)	26 (26.3)	73 (73.7)	0.902
* No*	172 (63.5)	44 (25.6)	128 (74.4)	
**Water treatment**				
* Yes*	14 (5.2)	3 (21.4)	11 (78.6)	1.000
* No*	257 (94.8)	67 (26.1)	190 (73.9)	
**Living near the stream**				
* Yes*	232 (85.6)	62 (26.7)	170 (73.3)	0.412
* No*	39 (14.4)	8 (20.5)	31 (79.5)	
**Draw water from the stream**				
* Yes*	49 (18.1)	10 (20.4)	39 (79.6)	0.338
* No*	222 (81.9)	60 (27.0)	162 (73.0)	
**Draw water from the tap**				
* Yes*	119 (43.9)	28 (23.5)	91 (76.5)	0.444
* No*	152 (56.1)	42 (27.6)	110 (72.4)	
**Disinfects drinking water**				
* Yes*	19 (7.0)	3 (15.8)	16 (84.2)	0.418
* No*	252 (93.0)	67 (26.6)	185 (73.4)	
**Washing clothes in streams**				
* Yes*	29 (10.7)	7 (24.1)	22 (75.9)	0.826
* No*	242 (89.3)	63 (26.0)	179 (74.0)	
**Swimming in streams**				
* Yes*	261 (96.3)	67 (25.7)	194 (74.3)	0.722
* No*	10 (3.7)	3 (30.0)	7 (70.0)	
**Haematuria**				
* Yes*	86 (31.7)	69 (80.2)	17 (19.8)	**< 0.0001**
* No*	185 (68.3)	1 (0.5)	184 (99.5)	
**RBCs**	4.5 (3.8, 5.0)	4.2 (3.4, 5.0)	4.5 (3.9, 5.0)	0.1221
**Haemoglobin g/dl**	11.8 (10.2, 13)	11.3 (8.3, 12.3)	11.9 (10.3, 13.5)	**0.0067**
**MCV**	83 (77, 88)	82 (78, 88)	83 (77, 88)	0.8754
**MCH**	26.3 (23.8, 28.1)	26.1 (23.9, 27.8)	26.4 (23.8, 28.2)	0.4123
**MCHC**	31.8 (30.5, 32.8)	30.9 (29.6, 32.6)	32.1 (30.8, 32.8)	**0.0022**
**Granulocytes**	2.9 (2.0, 4.5)	3.1 (2.0, 4.6)	2.9 (2.0, 4.4)	0.4516
**Lymphocytes**	1.9 (1.4, 2.4)	1.9 (1.4, 2.9)	1.9 (1.4, 2.3)	0.3243
**Monocytes**	0.3 (0.2, 0.4)	0.3 (0.2, 0.5)	0.3 (0.2, 0.4)	0.0628
**Platelets**	254 (194, 326)	265.5 (194, 335)	240 (194, 320)	0.1100
**Have you received deworming**				
* Yes*	139 (51.3)	5 (3.6)	134 (96.4)	**< 0.0001**
* No*	132 (48.7)	65 (49.2)	67 (50.7)	
**History of Schistosomiasis**				
* Yes*	71 (26.2)	7 (9.9)	64 (90.1)	**< 0.0001**
* No*	200 (73.8)	63 (31.5)	137 (68.5)	

**Abbreviations**: IQR, interquartile range; RBC, red blood cell; g/dL, grams per deciliter; MCV, mean corpuscular volume; MCH, mean corpuscular haemoglobin; MCHC, mean corpuscular haemoglobin concentration

Figure 2, compares schistosomiasis prevalence between males and females, showing a prevalence of 27.9% (95% CI: 21.4%–35.6%) among males and 23.0% (95% CI: 16.0%–31.6%) among females. This visual summary highlights that schistosomiasis burden was comparable between sexes, consistent with the statistical analysis showing no significant association (*p* = 0.370).

### Factors associated with schistosomiasis infection

Variables for logistic regression analysis were selected based on a significance level of *p* < 0.05 in the univariate analysis. Additionally, sex and age were included in the model as potential confounders due to their biological relevance. Multivariate logistic regression identified lack of deworming (OR: 37.8; 95% CI: 5.78–247.4; *p* < 0.0001), absence of haematuria (OR: 0.0014; 95% CI: 0.0001–0.011; *p* < 0.0001), and lower haemoglobin levels (OR: 0.69; 95% CI: 0.48–0.99; *p* = 0.044) as significant predictors of Schistosomiasis (Table 2). Although mean corpuscular haemoglobin concentration (MCHC) was significant in the univariate model (OR: 0.84; 95% CI: 0.74–0.95; *p* = 0.008), it was not significant in the multivariate model (*p* = 0.754). Likewise, a history of Schistosomiasis was significant in the univariate analysis (OR: 4.20; 95% CI: 1.82–9.69; *p* = 0.001), but this association did not persist in the multivariate model (*p* = 0.905). Age and sex were not statistically significant in either model (Table 2).

**Table 2:** Logistic regression of factors associated with schistosomiasis

	Univariate analysis		Multivariate analysis	
Variable	**OR (95% CI)**	**P value**	**OR (95% CI)**	**P value**
Age, Years	0.94 (0.82, 1.07)	0.389	1.38 (0.84, 2.25)	0.199
Sex				
* Male*	Ref	Ref	Ref	Ref
* Female*	0.77 (0.44, 1.35)	0.370	1.77 (0.40, 7.73)	0.442
Haematuria				
* Yes*	Ref	Ref	Ref	Ref
* No*	0.0013 (0.0001, 0.0102)	< 0.0001	0.0014 (0.0001, 0.011)	**< 0.0001**
HB	0.84 (0.75, 0.95)	**0.005**	0.69 (0.48, 0.99)	**0.044**
MCHC	0.84 (0.74, 0.95)	**0.008**	0.96 (0.76, 1.20)	0.754
Received deworming				
* Yes*	Ref	Ref	Ref	Ref
* No*	26 (9.99, 67.6)	< 0.0001	37.8 (5.78, 247.4)	**< 0.0001**
History of Schistosomiasis				
* Yes*	Ref	Ref	Ref	Ref
* No*	4.20 (1.82, 9.69)	**0.001**	1.12 (0.16, 7.72)	0.905

Abbreviations: OR, odds ratio; CI, confidence interval; RBC, red blood cell; HB, haemoglobin; MCHC, mean corpuscular haemoglobin concentration; Ref., reference category

## Discussion

This study aimed to determine the prevalence of schistosomiasis among school-aged children and adolescents visiting Chitokoloki Mission Hospital of Zambezi District. In addition, to identify the correlates of schistosomiasis among the selected group. The study findings revealed a schistosomiasis prevalence of 25.8%, which aligns with rates reported in South-western Nigeria (26.8%–29.5%) [25]. However, this was lower than the prevalence observed in other Nigerian studies (45.6%) [26] and in South Africa (75%) [27], yet higher than rates documented in Zambia which was conducted in Siavonga District of Southern Province and in Lusaka (9.7%) [20], and Mauritania (4.0%–15.6%) [28, 29].

These disparities may be attributed to differences in environmental and ecological conditions including high temperatures in North-western Province that creates a favourable condition for the spread of Schistosoma species. Warmer conditions accelerate the parasite’s life cycle by enhancing snail growth and reproduction in intermediate hosts such as Bulinus and Biomphalaria species [30]. Elevated temperatures also speed up the development of the parasite within snails, leading to increased release of infectious cercariae, while promoting longer larval survival in freshwater bodies [31].

In addition to temperature, specific ecological characteristics of Zambezi District, including the extensive Zambezi floodplain, seasonal flooding, slow-moving streams, and swampy wetland areas, support continuous breeding of Bulinus snails. These ecological drivers, unique to Northwestern Province, facilitate sustained transmission throughout the year and partly explain the high burden observed.

Social and behavioural factors play a major role in the continued spread of schistosomiasis, often worsening the effects of environmental risks [32]. Poverty limits access to clean water and sanitation, forcing communities to rely on rivers and streams for bathing, washing, and farming activities that increase direct contact with contaminated water [8]. Fishing and agricultural activities further heighten exposure, especially among male children and adolescents [33]. Poor sanitation practices such as open defecation and urination introduce parasite eggs into water bodies, sustaining transmission [2, 34]. Communities depending on freshwater for religious activities, household chores, and recreation face elevated risk, particularly during rainy seasons when water access is poor [35].

Another key finding from this study was that children who had not been dewormed with praziquantel were nearly 37.8 times (aOR: 37.8; 95%CI: 5.78, 247.4, *p* < 0.0001) more likely to be infected, highlighting the protective role of preventive chemotherapy. This observation is consistent with global evidence that regular deworming significantly reduces schistosomiasis prevalence and worm burden [36]. When interpreted in the national context, these findings suggest an important gap in schistosomiasis control in Zambia. Although national MDA coverage for parasitic infections is approximately 65%, schistosomiasis-specific coverage is far more variable, with some areas such as Ng’ombe in Lusaka Province reporting coverage as low as 49.8% [37]. This inconsistency indicates that many eligible children remain unreached, sustaining local transmission.

Low and inconsistent MDA coverage in Zambia is often driven by operational challenges such as drug shortages, limited transport and outreach to remote rural schools, inadequate community sensitization, and dependence on overburdened school health staff. In Northwestern Province, long distances between settlements, difficult terrain, and seasonal flooding further disrupt MDA outreach, reducing treatment uptake and contributing to persistent hotspots.

While praziquantel remains highly effective for clearing existing infections, it does not prevent reinfection. The drug’s mechanism action of action causes disruption of the parasite’s calcium homeostasis causing tegumental damage and limits its impact to current infections without protecting against future exposure [38, 39]. Repeated annual or biannual administration of praziquantel to at‑risk school‑aged children and community members in hyperendemic regions, as recommended by WHO, is therefore essential for sustained control [40]. Moreover, integrating schistosomiasis control with broader WASH interventions, including improved access to safe water, sanitation facilities, and hygiene education would provide a more sustainable approach to reducing transmission.

The absence of haematuria showed a strong protective effect of schistosomiasis infection. This finding contrasts with urogenital schistosomiasis, in which this marker is well established as a specific clinical indicator of active infection, aligning with WHO diagnostic guidelines and other studies [41, 42]. In resource‑constrained environments lacking access to standard schistosomiasis assays, the detection of haematuria constitutes a sensitive and prognostically informative biomarker for active infection.

Lower haemoglobin levels were significantly associated with infection likely reflecting chronic blood loss or inflammation-induced anaemia caused by *S. haematobium*. This finding aligns with evidence from Degarege et al., and Leir et al. who documented chronic blood loss, tissue damage, and inflammatory processes as key drivers of iron-deficiency anaemia in infected populations [43–49]. These physiological manifestations highlight the broader nutritional and haematological impact of schistosomiasis and reinforce the need for integrated anaemia management within control programs.

These findings have important implications for school-based and community-based interventions. Schools provide an ideal platform for monitoring treatment uptake, maintaining accurate deworming records, and implementing termly urine dipstick screening in high-risk areas. School-based health education focusing on reducing unnecessary water contact, promoting consistent latrine use, and encouraging early treatment-seeking could significantly reduce risk. Integrating schistosomiasis education into the school curriculum and involving parent–teacher associations would strengthen community participation and behaviour change.

At community level, identifying villages with high water-contact behaviours, mapping known human–water interaction points, and prioritizing outreach to floodplain settlements would improve targeted surveillance. Community Health Assistants can support routine household-level screening, health education, and reporting of suspected cases. School absenteeism often associated with chronic schistosomiasis could serve as an additional surveillance indicator for early identification of clusters of infection.

A key strength of this study lies in its integration of both parasitological and haematological markers to improve diagnostic accuracy, along with a multivariable logistic regression model that controlled for key demographic, behavioural, and clinical confounders. These elements enhanced the reliability of the findings.

This study has several limitations that must be considered when interpreting the findings. First, the retrospective and cross-sectional design restricts causal inference, meaning that the temporal relationship between schistosomiasis and associated factors cannot be established. Second, because the sample was derived exclusively from hospital records, it may over-represent symptomatic or unwell children, resulting in sampling and selection bias and a likely overestimation of prevalence compared to what would be observed in community-based surveys. Third, data completeness was a significant challenge, as 45% (339/610) of records were excluded due to missing variables, introducing potential selection bias; although limited comparison of available characteristics (age, sex, haematuria) showed no major differences between included and excluded records, this still reduces the generalisability of findings. Fourth, several predictors in the logistic regression model exhibited extremely wide confidence intervals for example, the adjusted odds ratio for lack of deworming (aOR = 37.8; 95% CI: 5.78–247.4), reflecting sparse data and instability within certain categories, underscoring the need for larger and more balanced samples in future research.

## Conclusion

This study highlights a significant burden of urogenital schistosomiasis among children in Zambezi District, underscoring the important need to strengthen praziquantel outreach and address operational gaps in MDA delivery. This should include routine school-based haematuria screening, enhanced health education on safe water practices, and targeted community surveillance particularly in floodplain communities and should be integrated into district control strategies. Implementing these child-focused interventions can accelerate progress toward Zambia’s schistosomiasis elimination targets and reduce long-term morbidity.

## Data Availability

The datasets used and/or analysed during the current study are available from the corresponding author on reasonable request.

## Abbreviations

LMICs: Low–and Middle–Income Countries
MDA: Mass drug administration
RBCs: Red blood cells
SSA: Sub–Saharan Africa
WASH: Water, Sanitation, and Hygiene
WHO: World Health Organization
